# Binary-phase acoustic passive logic gates

**DOI:** 10.1038/s41598-019-44769-0

**Published:** 2019-06-07

**Authors:** Yin Wang, Jian-ping Xia, Hong-xiang Sun, Shou-qi Yuan, Xiao-jun Liu

**Affiliations:** 1 Research Center of Fluid Machinery Engineering and Technology, Faculty of Science, Jiangsu University, Zhenjiang, 212013 China; 2 Key Laboratory of Modern Acoustics, Department of Physics and Collaborative Innovation Center of Advanced Microstructures, Nanjing University, Nanjing, 210093 China; 3 State Key Laboratory of Acoustics, Institute of Acoustics, Chinese Academy of Sciences, Beijing, 100190 China

**Keywords:** Acoustics, Materials for devices

## Abstract

The recent rapid development of acoustic logic devices has opened up the possibilities of sound computing and information processing. However, simultaneous realization of acoustic logic devices with subwavelength size, broad bandwidth and passive structure still poses a great challenge. To overcome it, we propose a subwavelength acoustic logic gate which consists of binary-phase passive unit cells placed into a multi-port waveguide. Based on the phase manipulations of the unit cells, we experimentally and numerically realize three basic logic gates OR, NOT and AND, and a composite logic gate XOR with a uniform threshold of 0.4 Pa based on linear acoustic interferences. More importantly, We also design a composite logic gate XNOR by a four-port waveguide, and composite logic gates NOR and NAND and a logic operation A⊙(B+C) based on two logic gates. We demonstrate a 0.6λ-length, 0.3*λ-*width, and 0.2-fractional bandwidth acoustic logic gate constructed by passive structures, which may lead to important advances in various applications, such as acoustic computing, acoustic information processing and integrated acoustics.

## Introduction

As major critical components in modern electronic integrated circuits, Boolean logic gates have attracted more and more attentions owing to its capability of implementing Boolean functions. Motivated by great potentials in next-generation photonic integrated circuits, the Boolean logic gates have been introduced in the field of optics^1–9^. By using photonic crystals^2^, nanophotonic plasmon networks^4^, and metal slot waveguide networks^5,6^, optical logic gates have been realized based on linear interferences of input and control signals with a certain phase difference^9^.

Inspired by the optical logic gates, the design of acoustic logic devices has also become a research hotspot owing to a variety of important potential applications^10–17^, such as acoustic computing, acoustic information processing and integrated acoustics. Based on a nonlinear dynamical effect of a driven granular chain composed of spherical particles, Li *et al*.^13^ have experimentally realized an acoustic switch and logic gates AND and OR. Beyond that, the rapid development of sonic crystals (SCs)^18–22^ and acoustic metamaterials (AMMs)^23–29^ has provided feasibility for realizing linear acoustic logic devices. By using the SCs, Bringuier *et al*.^30^ have designed logic gates NAND, XOR and NOT based on the linear interferences of input and control signals. Zhang *et al*.^31^ have theoretically proposed and experimentally demonstrated basic Boolean logic gates XOR, OR, AND and NOT by using self-collimated beams in the SCs with line-defects. However, the acoustic logic devices fabricated with the SCs inevitably involve large structure size at low frequency. By introducing density-near-zero AMMs^32–34^, a series of Boolean logic gates AND, OR, XOR and NOT and a logic operation have been realized based on two input signals with a certain phase difference^35^. Additionally, as a category of AMMs, acoustic metasurfaces with the characteristics of ultrathin planar structure and wholly controlled phase delay^36–40^ have been introduced to design acoustic analog computing systems for *n*th-order ordinary differential equations^41^. Moreover, the recent emerging acoustic topological insulators^42–47^ immune to backscattering has great significance for the development of acoustic logic devices. As an example, Xia *et al*.^48^ have proposed acoustic topological insulators with reconfigurable and programmable functionalities realize two types of acoustic switches and tunable logic gates OR and XOR. Furthermore, to realize acoustic logic gates with broad bandwidth, Zuo *et al*.^49^ have designed an acoustic logic gate based on multi-port circular waveguides, in which its working bandwidth is larger than 5 kHz and all logic functions and a complex logic operation can be realized by modulating signal phases. The aforementioned works have realized acoustic logic gates and operations with high efficiency. However, most of them have been designed based on active manipulations of the phases of input and control signals, and their structure sizes are still large, which severely limits their integration and practical applications. Simultaneous realization of acoustic logic devices with subwavelength size, broad bandwidth and passive structure still poses a great challenge.

In this work, we propose a subwavelength acoustic logic gate which consists of binary-phase passive unit cells placed into a multi-port waveguide. Based on the phase manipulations of the unit cells, we design the basic logic gates OR, NOT and AND, and two composite logic gates XOR and XNOR with a uniform threshold of 0.4 Pa based on the linear acoustic interferences, and the fractional bandwidth (the ratio of the bandwidth to the center frequency) can reach about 0.2. The measured results agree well with the simulated ones. Finally, we also present the realizations of two composite logic gates NOR and NAND and a logic operation A⊙(B+C) based on two logic gates.

## Results and Discussions

### Design of acoustic logic gate

**Figure 1:**
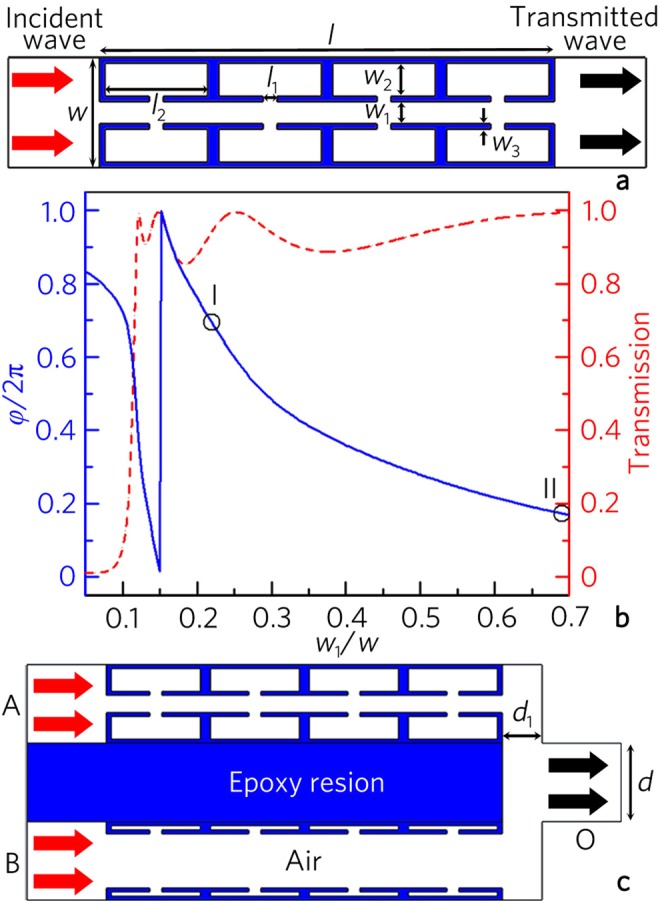
Design of acoustic logic gate. (**a**) Schematic of a unit cell of phase manipulation based on two symmetric Helmholtz resonator arrays. (**b**) Phase delays (blue solid line) and transmissions (red dashed line) created by unit cells with different width ratios *w*_1_/*w*. (**c**) Schematic of acoustic logic gate. Red arrows at ports A and B represent same input signals, and black arrows are output signals.

As shown in Fig. 1a, the unit cell (with length *l* = *λ*/2 and width *w* = *λ*/10) consists of two symmetric Helmholtz resonator arrays and a straight channel^50^ with a tunable width *w*_1_, where λ is the wavelength. The cavity width *w*_2_ is determined by *w*_1_, and the other parameters *l*_1_ = 0.03 *l*, *l*_2_ = 0.225 *l*, *w*_3_ = 0.05*w* and *w*_2_ = *w − w*_1_ − 4*w*_3_. The unit cell filled with air is fabricated with epoxy resin to satisfy the sound hard boundary condition. The wavelength *λ* of input acoustic signals is selected as 10 cm (viz., frequency *f*_0_ = 3430 Hz in air).

Figure 1b shows the phase delays (blue solid line) and transmissions (red dashed line) created by the unit cells with different width ratios (*w*_1_/*w*). Note that, in the range 0.12–0.70, the phase delay of the unit cell can cover the whole 2π range, and the transmission is larger than 0.85. Here, the two selected unit cells I and II (black hollow dots) with the parameter *w*_1_/*w* of 0.22 and 0.69, which correspond to the transmissions *T*_I_ = 0.94 and *T*_II_ = 0.99 and the difference of the phase delays *φ*_I_ − *φ*_II_ = π, are used to design acoustic logic gates.

As shown in Fig. 1c, we design an acoustic logic gate which consists of a symmetric three-port waveguide with a rectangular solid made of epoxy resins and two types of passive unit cells placed into two channels, in which the parameters *d* = 0.1*λ* and *d*_1_ = 0.055*λ*. Note that the width and length of the logic gate structure are only about 0.3*λ* and 0.6*λ*, respectively. When the unit cells in the channels A and B are different (shown in Fig. 1c), two output signals with out-phase characteristic produce the interference cancellation at the port O. However, the interference enhancement is obtained at the port O by placing the same unit cells into the channels A and B owing to the in-phase characteristic of output signals. Based on the linear acoustic interferences, we can realize all acoustic logic gates and complex logic operations.

### Logic OR and XOR gates

**Figure 2:**
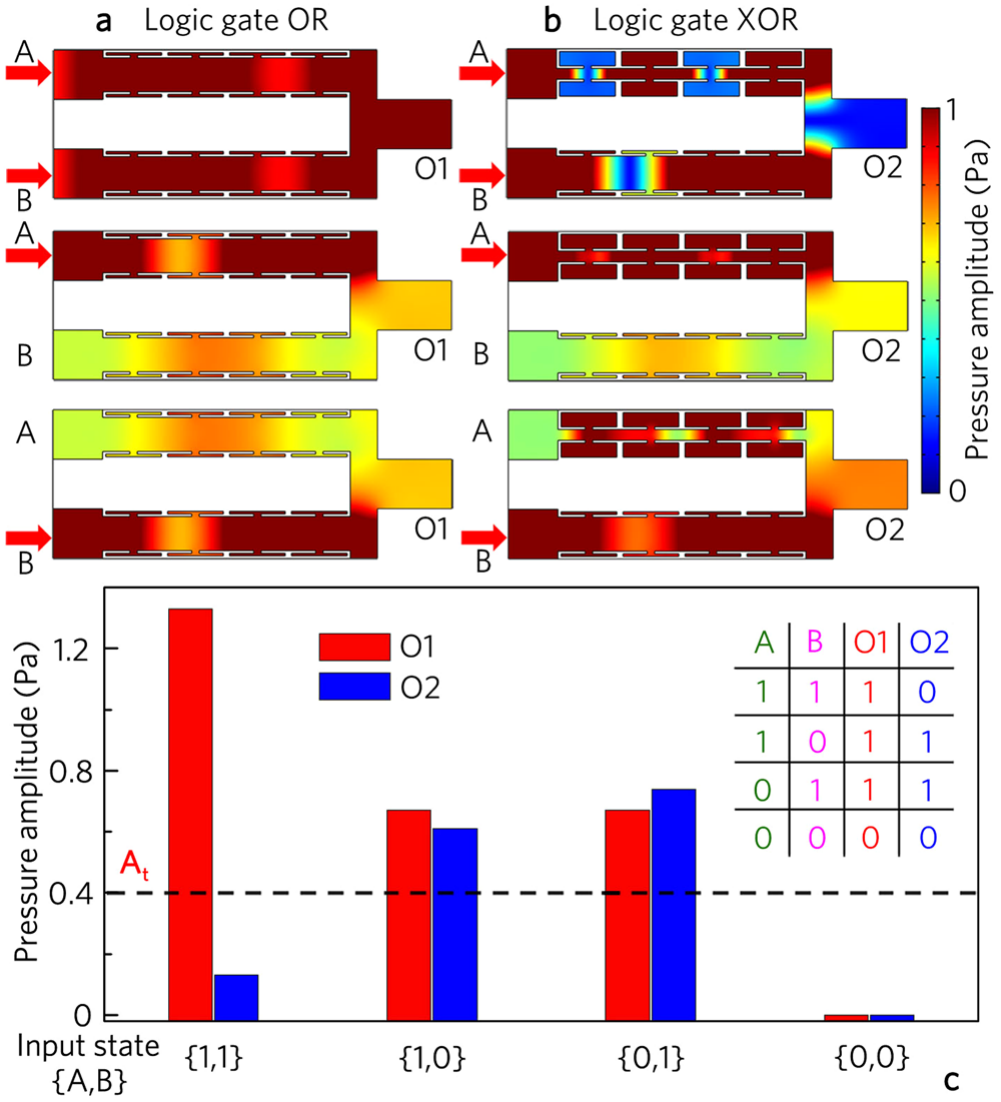
Logic OR and XOR gates. Distributions of pressure amplitude field induced by logic gates (**a**) OR and (**b**) XOR for different input states, and (**c**) corresponding output pressure amplitudes and true table.

Figure 2 shows the simulated results for the logic gates OR and XOR, in which the unit cells II is placed into both channels A and B for the logic gate OR, but the unit cells I and II are used to design the logic gate XOR. Here, the same incident signals (with the initial amplitude of 1.0 Pa) are placed at the input ports A and B. The input state is expressed as {*Q*_1_, *Q*_2_}, in which *Q*_i_ represent if there is (encode “1”) or not (encode “0”) acoustic signal from the respective input port. In addition, to determine the output states, we introduce a uniform threshold A_t_ = 0.4 Pa. When the output amplitude is larger than *A*_t_, the output state is 1/TRUE, while the output is 0/FALSE with the output amplitude smaller than A_t_.

As shown in Fig. 2a,b, the output amplitude for the input state {1, 1} is about two times larger than those for the input states {1, 0} and {0, 1} in the logic gate OR (Fig. 2a), while that is almost zero for the input state {1, 1} in the logic gate XOR (Fig. 2b). Such a phenomenon is attributed to the interference enhancement and cancellation at the output port O owing to the phase manipulation of the unit cells, which is different from previous works based on external active manipulations of signal phases^31,35,49^. Figure 2c shows the bar charts of the output amplitudes and the true table of the logic gates OR and XOR. By introducing the uniform threshold of 0.4 Pa, the output states are {1}, {1}, {1} and {0} for the input states {1, 1}, {0, 1}, {1, 0} and {0, 0} at the port O1, and {0}, {1}, {1} and {0} at the port O2, realizing the logic gates OR and XOR, respectively.

### Logic NOT gate

**Figure 3:**
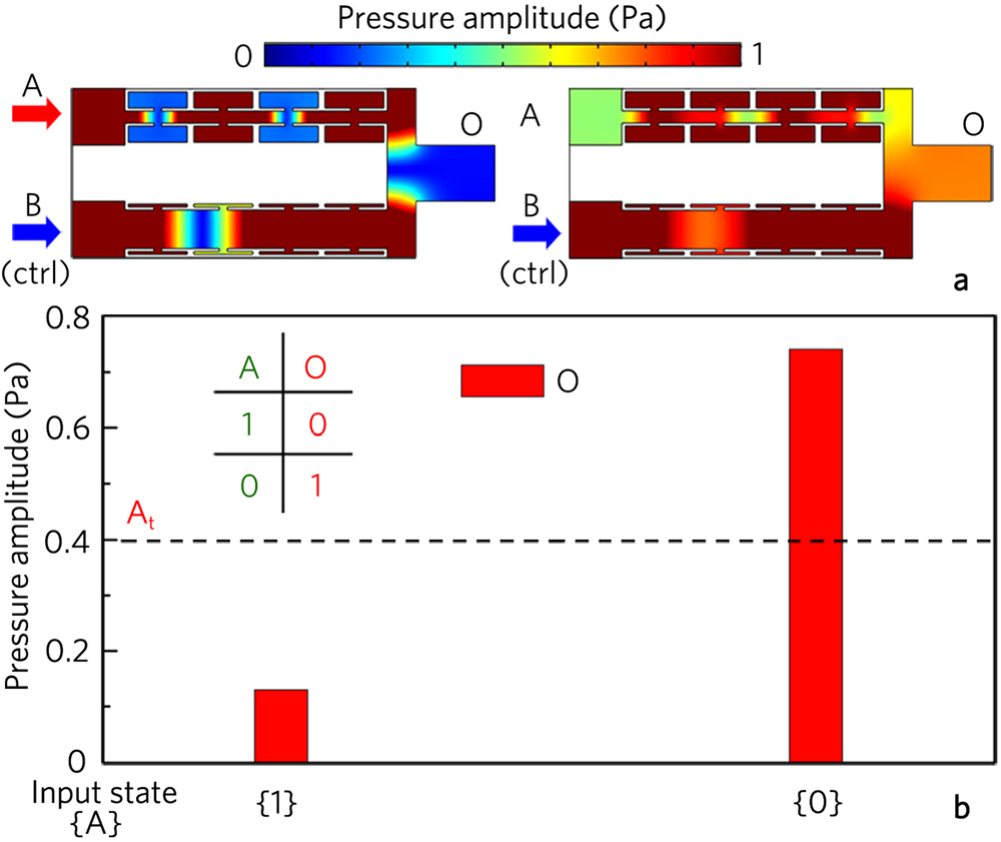
Logic NOT gate. (**a**) Distributions of pressure amplitude field induced by logic gate NOT for different input states, and (**b**) corresponding output pressure amplitudes and true table.

We also realize the logic gate NOT with the same structure as the logic gate XOR, in which A is the input port, and B is the control port with a constant signal. As shown in Fig. 3a, the output amplitude approaches zero for the input state {1} owing to the interference cancellation, while that is larger than 0.7 Pa for the input state {0}. With the uniform threshold of 0.4 Pa, the corresponding output states are {0} and {1} for the input states {1} and {0} (Fig. 3b), respectively, realizing the logic gate NOT. The corresponding input and output states are also clearly shown in the truth table.

### Logic AND gate

**Figure 4:**
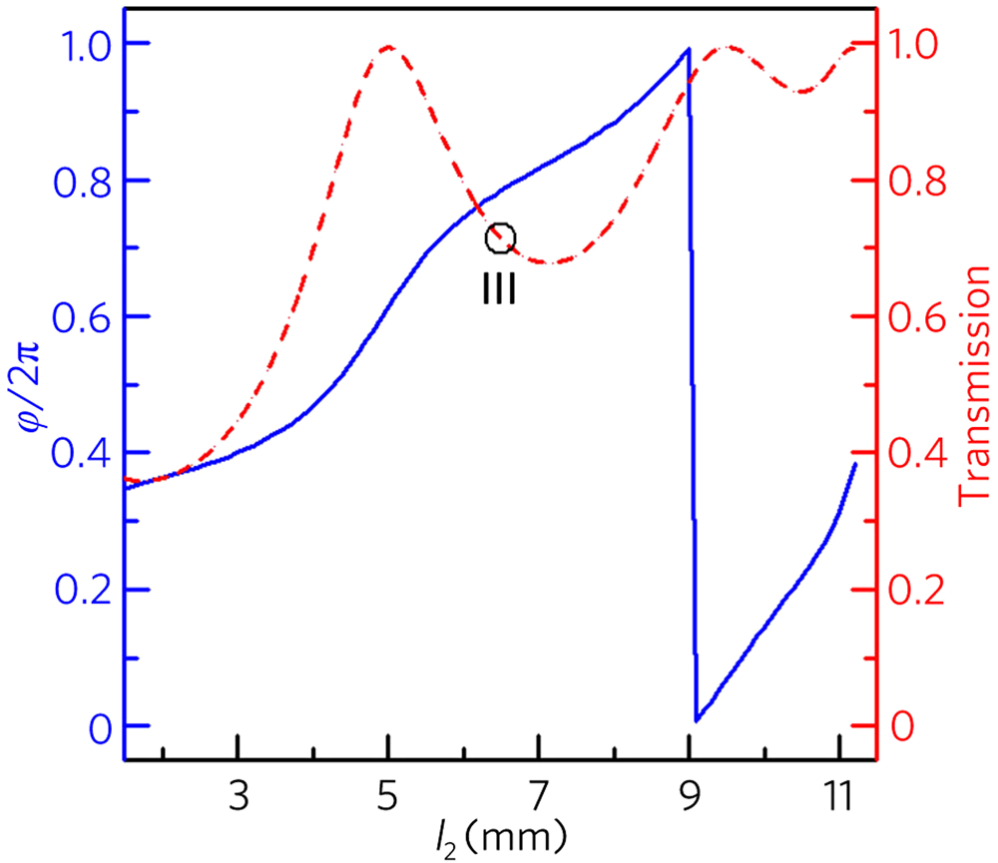
Performances of unit cell III. Phase delays (blue solid line) and transmissions (red dotted line) with different cavity widths *l*_2_.

In addition to the logical gates constructed by the three-port waveguide structure, we design the logic gate AND based on a four-port waveguide structure, in which the rectangular solid at the center of the three-port waveguide (Fig. 1) is replaced by the channel C with the same size as the channel A. To realize the logic function AND, we introduce another passive unit cell placed into the channel C as the control port. Figure 4 shows the phase delays (blue solid line) and transmissions (red dashed line) of the unit cell with different cavity widths (*l*_2_), in which the parameter *w*_1_/*w* is a constant of 0.12. Here, we select the unit cell III (the parameters *w*_1_/*w* = 0.12 and *l*_2_ = 6.5 mm) with the transmission *T*_III_ = 0.71 and the phase delay *φ*_III_ = *φ*_I_ + π/6, which is utilized to realize the output amplitude less than 0.4 Pa for the input state {0, 0} and the interference cancellation between the input and control ports.

**Figure 5:**
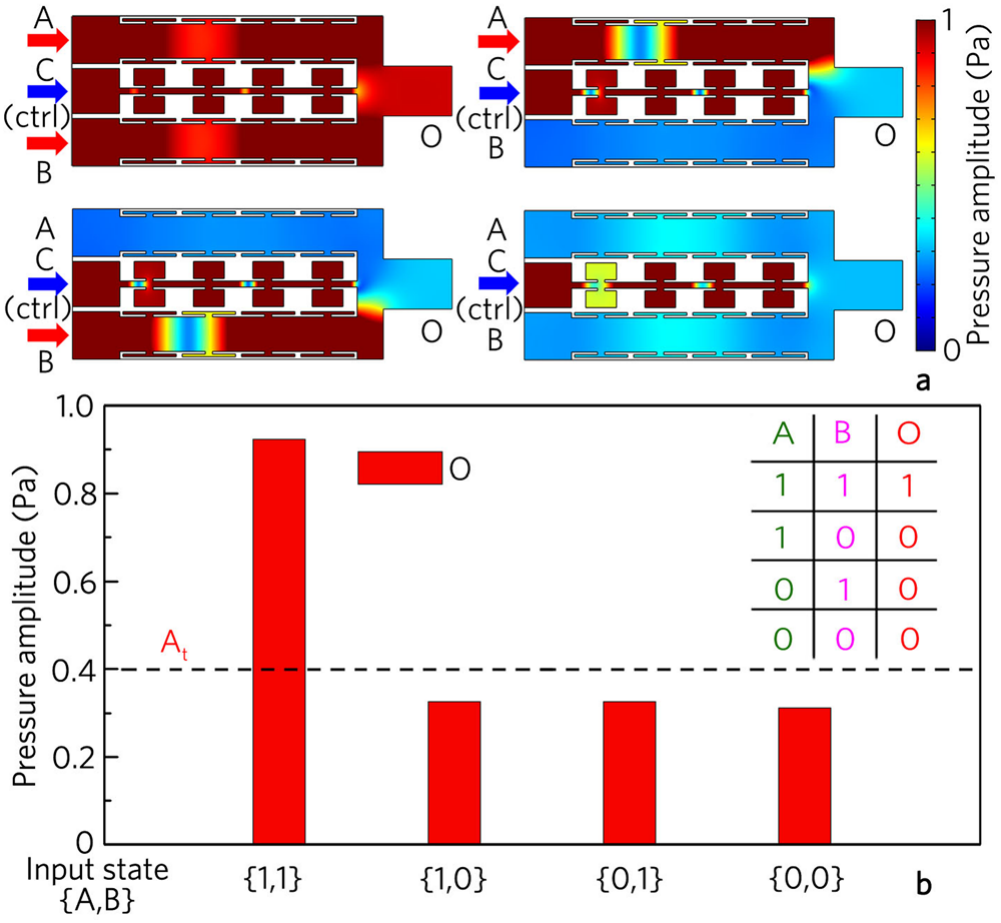
Logic AND gate. (**a**) Distributions of pressure amplitude field induced by logic gate AND for different input states, and (**b**) corresponding output pressure amplitudes and true table.

As shown in Fig. 5a, the output amplitude is about 0.92 Pa for the input state {1, 1}, which is smaller than that of the logic gate OR (Fig. 2a). Such energy reduction arises from the weak interference cancellation induced by the control signal from the port C. Moreover, the output amplitudes are smaller than 0.4 Pa for the input states {0, 1}, {1, 0} and {0, 0}, which is attributed to the interference cancellation induced by the signals from the port C and the ports A and B for {1, 0} and {0, 1}, and the sound energy propagating from the control port C to the other three ports for {0, 0}. Therefore, with the uniform threshold of 0.4 Pa, the output states are {1}, {0}, {0} and {0} for different input states (Fig. 5b), realizing the logic gate AND.

### Working bands of basic logic gates

**Figure 6:**
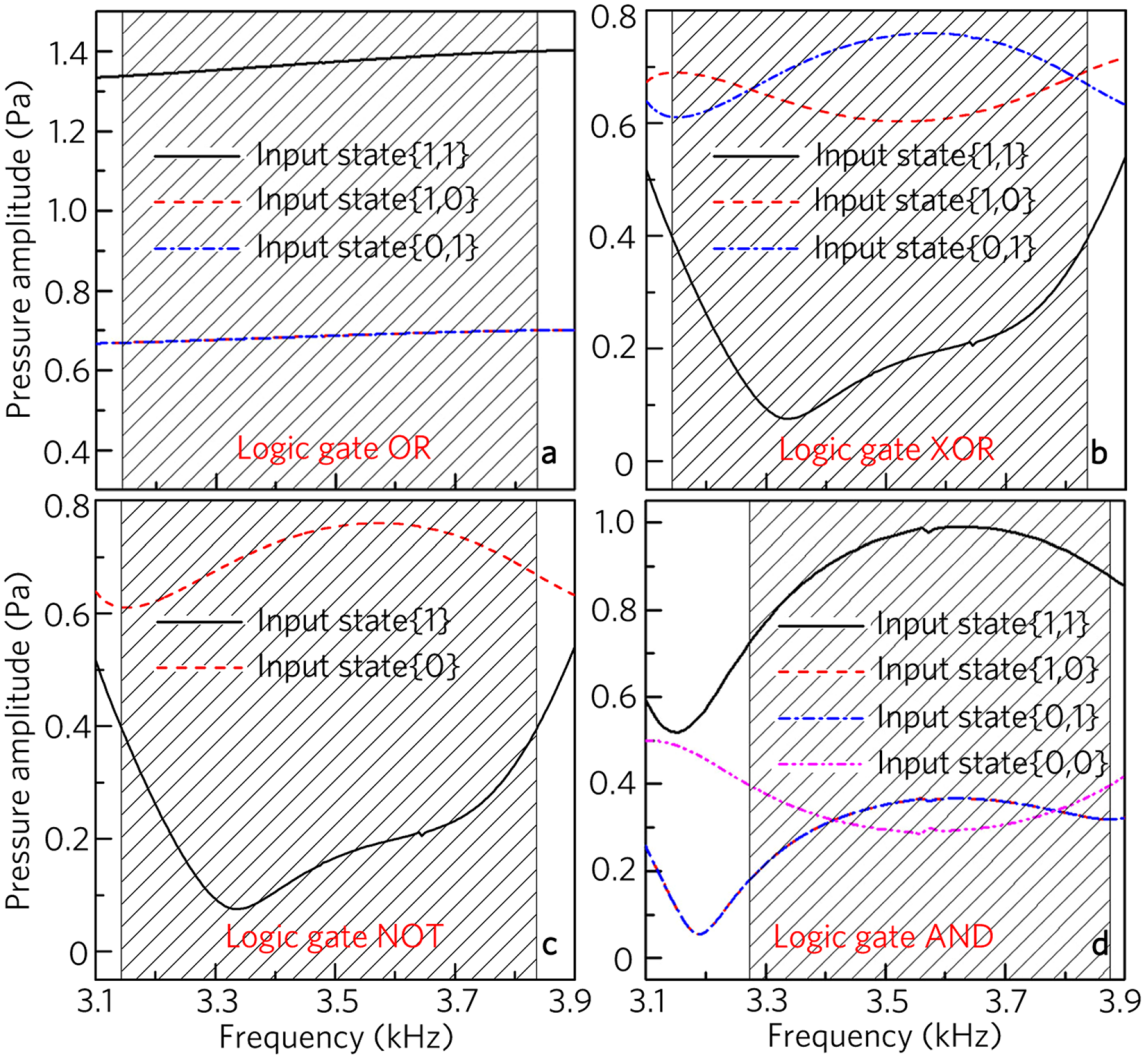
Working bands of basic logic gates. Output amplitude spectra induced by logic gates (**a**) OR, (**b**) XOR, (**c**) NOT and (**d**) AND for different input states.

The proposed logic gates have broadband characteristic. As shown in Fig. 6a, for the logic gate OR, the output amplitudes for the three input states are larger than 0.4 Pa, and its working band far exceeds the range 3140–3840 Hz (shadowed region) owing to the interference enhancement based on the phase manipulation of two same unit cells. However, the working bands are fixed in the ranges 3140–3840 Hz for the logic gates XOR (Fig. 6b) and NOT (Fig. 6c) and 3270–3880 Hz for the logic gate AND (Fig. 6d). This is because the design mechanisms are closely related to the interference cancellation based on two different unit cells with the out-phase characteristic, and the phase difference of the two unit cells is determined by the frequency. Therefore, we have demonstrated the broad bandwidth of the logic gates, in which the fractional bandwidth can reach about 0.2 for the logic gates OR, XOR and NOT, and is 0.17 for the logic gate AND.

### Experimental measurements

**Figure 7:**
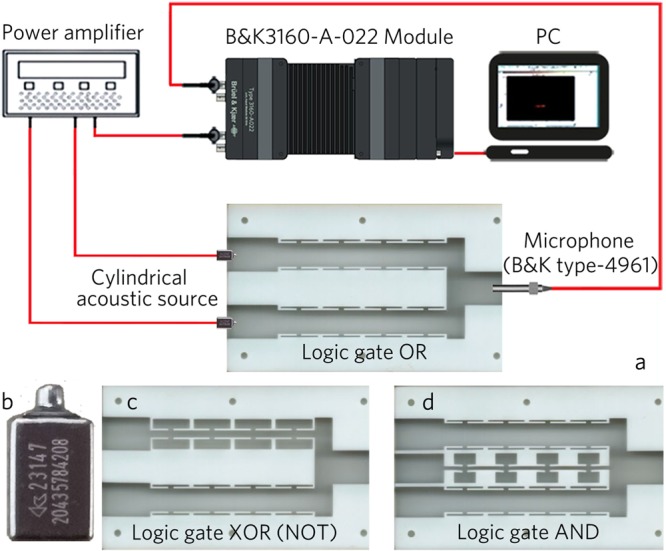
Experimental setup and sample photographs. (**a**) Experimental setup and photograph of logic gate OR. Photographs of (**b**) cylindrical acoustic source, (**c**) logic gate XOR (NOT) and (**d**) logic gate AND.

To further demonstrate the aforementioned logic gates, we experimentally measure output time-domain signals for these logic gates. The measurement setup and the photograph of the sample are shown in the Fig. 7.

**Figure 8:**
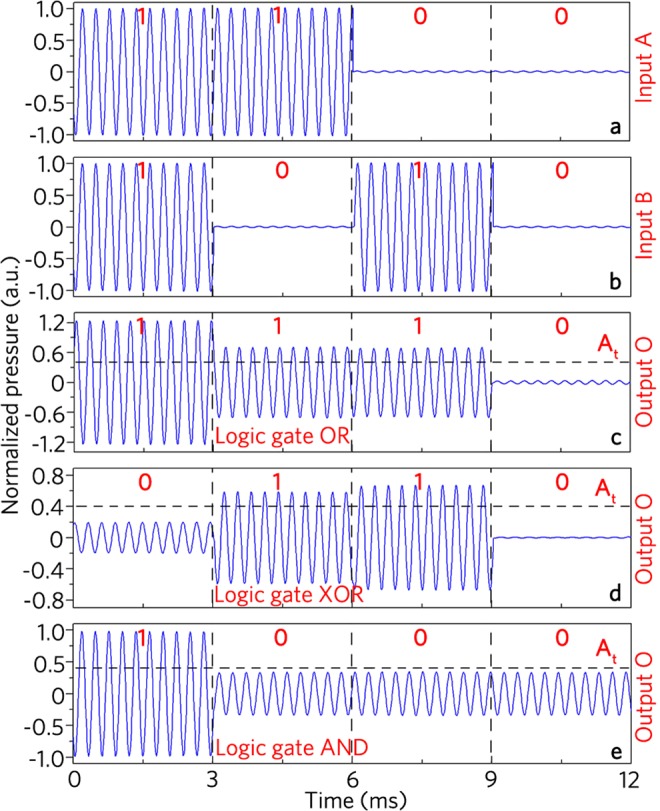
Measured time-domain signals. The input ports (**a**) A and (**b**) B and output port O in logic gates (**c**) OR, (**d**) XOR, and (**e**) AND.

Figure 8a-e shows the measured time-domain signals at the input ports A and B and the output port O for the logic gates OR, XOR and AND, respectively, in which all measured signals are normalized by the maximum amplitude in Fig. 8a. As shown in Fig. 8a,b, the input signals with the same initial phases at the ports A and B are modulated to realize four input states {1, 1}, {1, 0}, {0, 1} and {0, 0} in the time domains *t* = 0–3 ms, 3–6 ms, 6–9 ms and 9–12 ms, respectively. Figure 8c-e shows the measured output signals at the port O for the logic gates OR, XOR and AND, respectively. With the uniform threshold of 0.4 Pa, the measured output states for different input states are {1}, {1}, {1} and {0} in Fig. 8c; {0}, {1}, {1} and {0} in Fig. 8d; {1}, {0}, {0} and {0} in Fig. 8e, which agrees well with the logic functions OR, XOR and AND, respectively.

**Figure 9:**
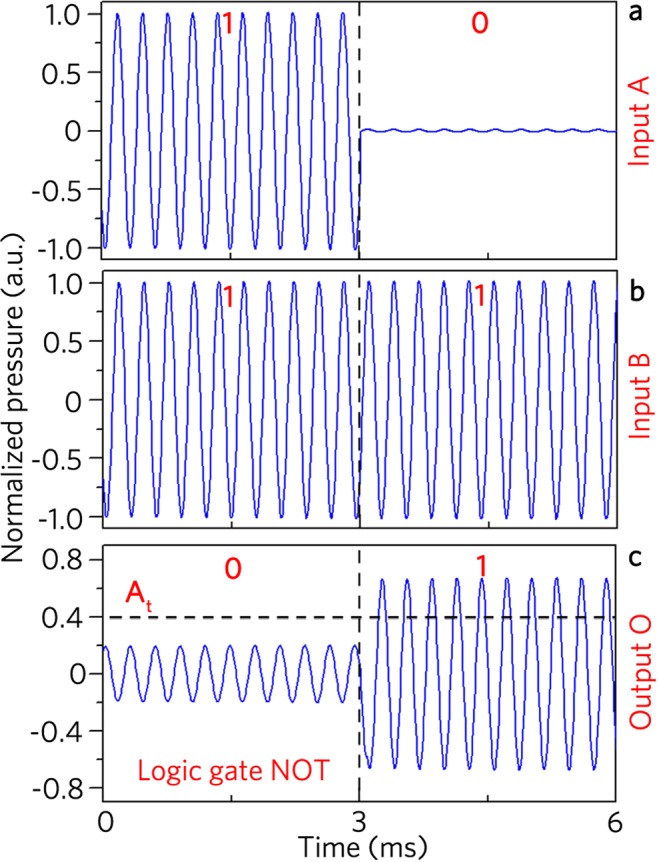
Measured time-domain signals. The input ports (**a**) A and (**b**) B and (**c**) output port O in logic gate NOT.

Moreover, as shown in Fig. 9a,b, the input signal at the port A is modulated to realize two input states {1} and {0} in the time domains *t* = 0–3 ms and 3–6 ms, respectively, and the constant signal placed at the port B acts as a control port (Fig. 9b). Figure 9c shows the measured output signals for the logic gate NOT, in which the output states are {0} and {1} based on the uniform threshold of 0.4 Pa, and the experiment results agree well with the logic function NOT. However, there exist weak output signals for the input states {1, 1} (Fig. 8d) and {1} (Fig. 9c), which arises from non-complete interference cancellation of two signals induced by considerable fabrication errors of the passive unit cells. Therefore, we have experimentally demonstrated the logic gates OR, XOR, AND and NOT.

### Logic XNOR gate

**Figure 10:**
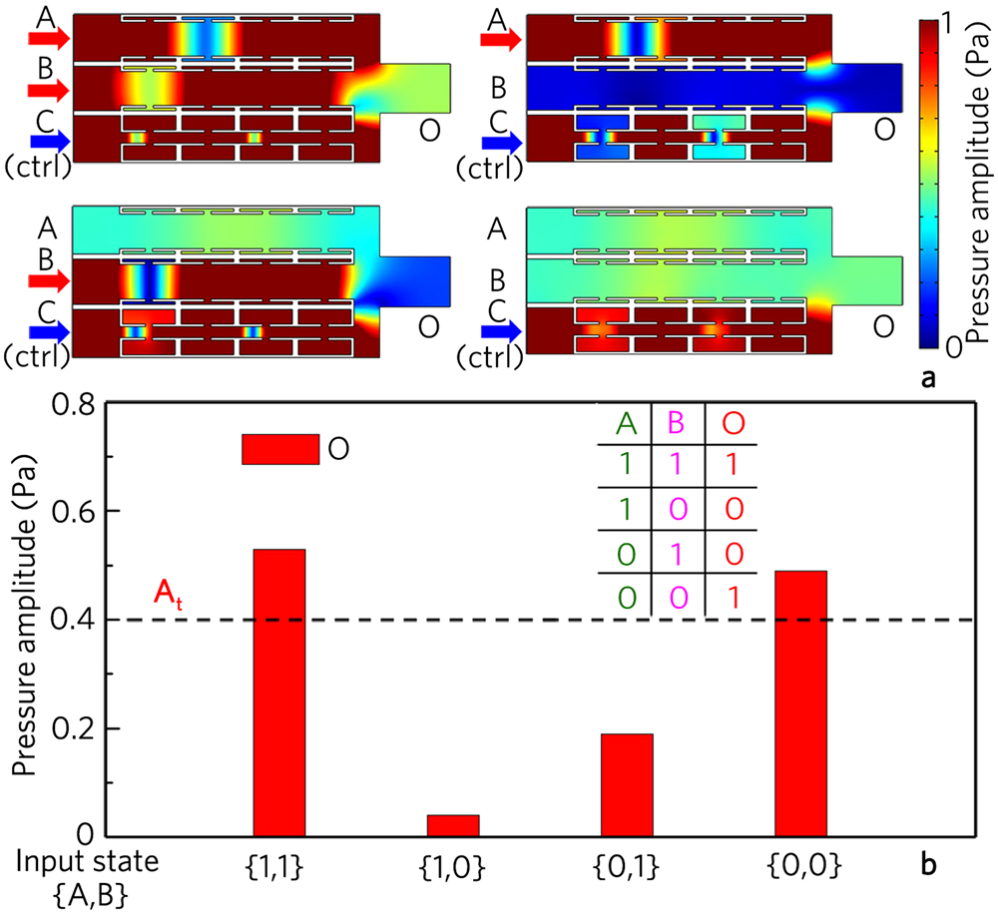
Logic XNOR gate. (**a**) Distributions of pressure amplitude field induced by logic gate XNOR for different input states, and (**b**) corresponding output pressure amplitudes and true table.

We can design the logic gate XNOR based on the four-port waveguide, in which the unit cell II is placed into the channels A and B as the input ports, but the unit cell I is placed into the channel C as the control port. As shown in Fig. 10a, the output amplitude at the port O for the input state {1, 1} is almost the same as that for {0, 0}, which arises from the fact that the interference cancellation exists between the signals from the ports A and C for {1, 1}, and therefore the output signals are from the ports B and C for the input states {1, 1} and {0, 0}, respectively. In addition, the output amplitudes are smaller than 0.4 Pa for the input states {1, 0} and {0, 1} owing to the interference cancellation of the out-phase signals created by the phase manipulation of the unit cells. As shown in Fig. 10b, with the uniform threshold of 0.4 Pa, the output states are {1}, {0}, {0} and {1} for the input states {1, 1}, {0, 1}, {1, 0} and {0, 0}, respectively, which is also clearly shown in the truth table. Therefore, the logic gate XNOR is realized.

### Logic NOR gate

**Figure 11:**
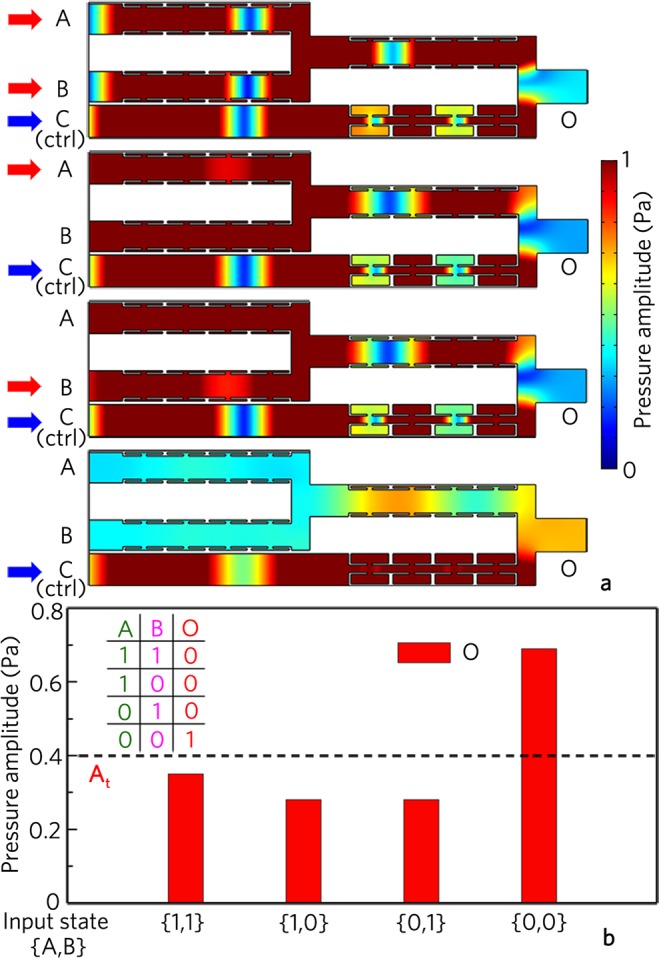
Logic NOR gate. (**a**) Distributions of pressure amplitude field induced by logic gate NOR for different input states, and (**b**) corresponding output pressure amplitudes and true table.

Owing to the easy connection characteristic of the multi-port waveguide, we can realize the composite logic gates NOR and NAND based on two basic logic gates. Figure 11 shows the simulated results for the composite logic gate NOR composed of the logic gates OR and NOT, in which A and B are the input ports, and C is the control port. As shown in Fig. 11a, the output amplitude for the input state {0, 0} at the port O is about 0.7 owing to the signal energy from the control port C. However, the output amplitudes are smaller than 0.4 Pa for the input states {1, 1}, {0, 1}, and {1, 0}, which is attributed to the interference cancellation induced by two signals from output port of the logic gate OR and the control port C. As shown in Fig. 11b, with the uniform threshold of 0.4 Pa, the output states are {0}, {0}, {0} and {1} for the input states {1, 1}, {1, 0}, {0, 1} and {0, 0}, respectively. Therefore, we have realized the logic gate NOR based on the basic logic gates OR and NOT.

### Logic NAND gate

**Figure 12:**
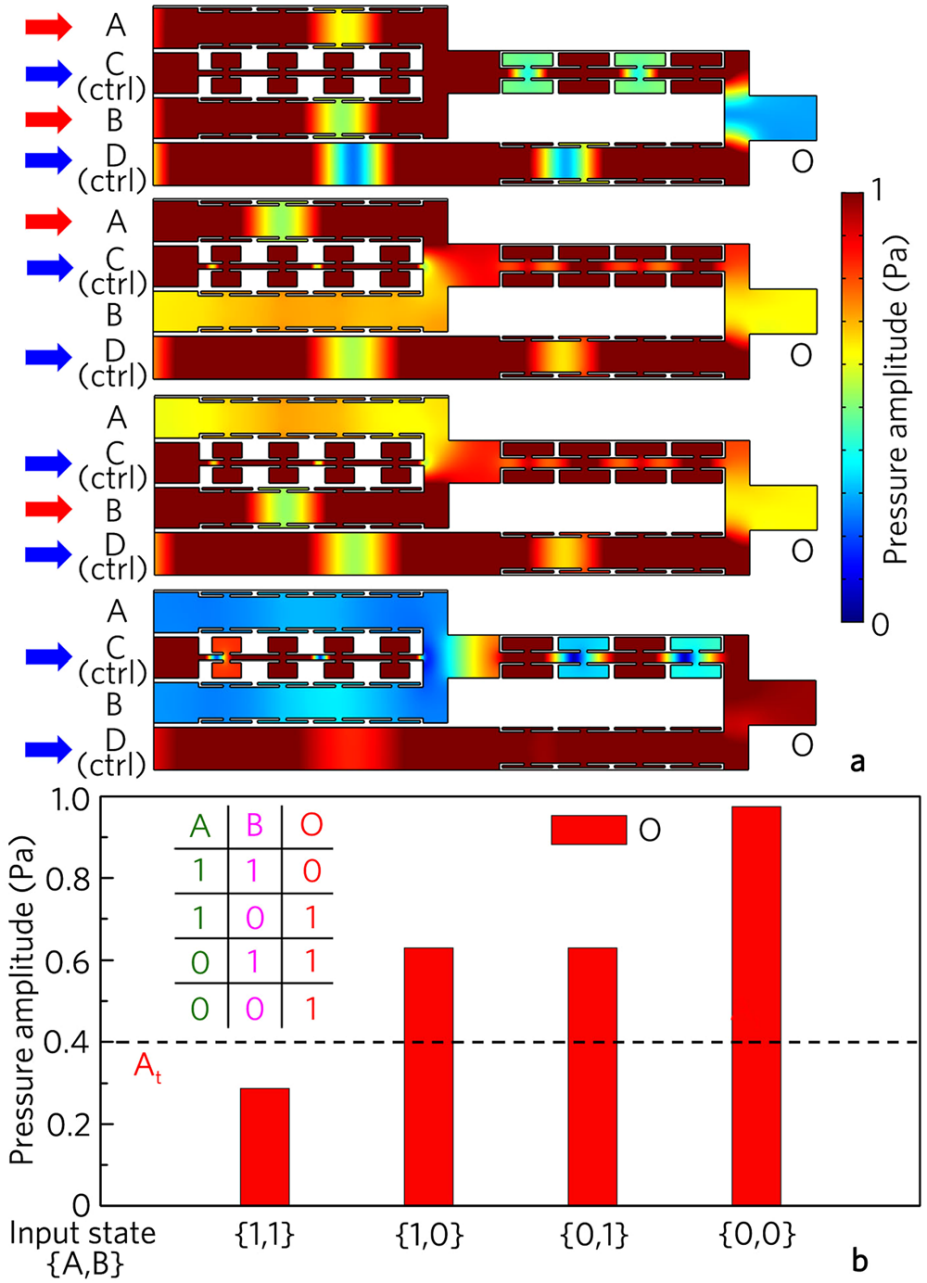
Logic NAND gate. (**a**) Distributions of pressure amplitude field induced by logic gate NAND for different input states, and (**b**) corresponding output pressure amplitudes and true table.

Furthermore, as shown in Fig. 12, the composite logic gate NAND consists of the basic logic gates NOT and AND, in which A and B are the input ports, and C and D are the control ports. It is noted that the output amplitude for the input state {1, 1} is small than 0.4 Pa, which stems from the interference cancellation between the output signal of the logic gate AND and the control signal at the port D. Besides, the output amplitudes are large than 0.4 Pa for the input states {0, 1}, {1, 0} and {0, 0}. This is because the signal energy at the port D is stronger than that at the output port of the logic gate AND, and thus the weak interference cancellation is obtained at the port O. Thus, as shown in Fig. 12b, the output states are {0}, {1}, {1} and {1} for the input states {1, 1}, {1, 0}, {0, 1} and {0, 0}, respectively, realizing the logic gate NAND.

### Acoustic logic operation

**Figure 13:**
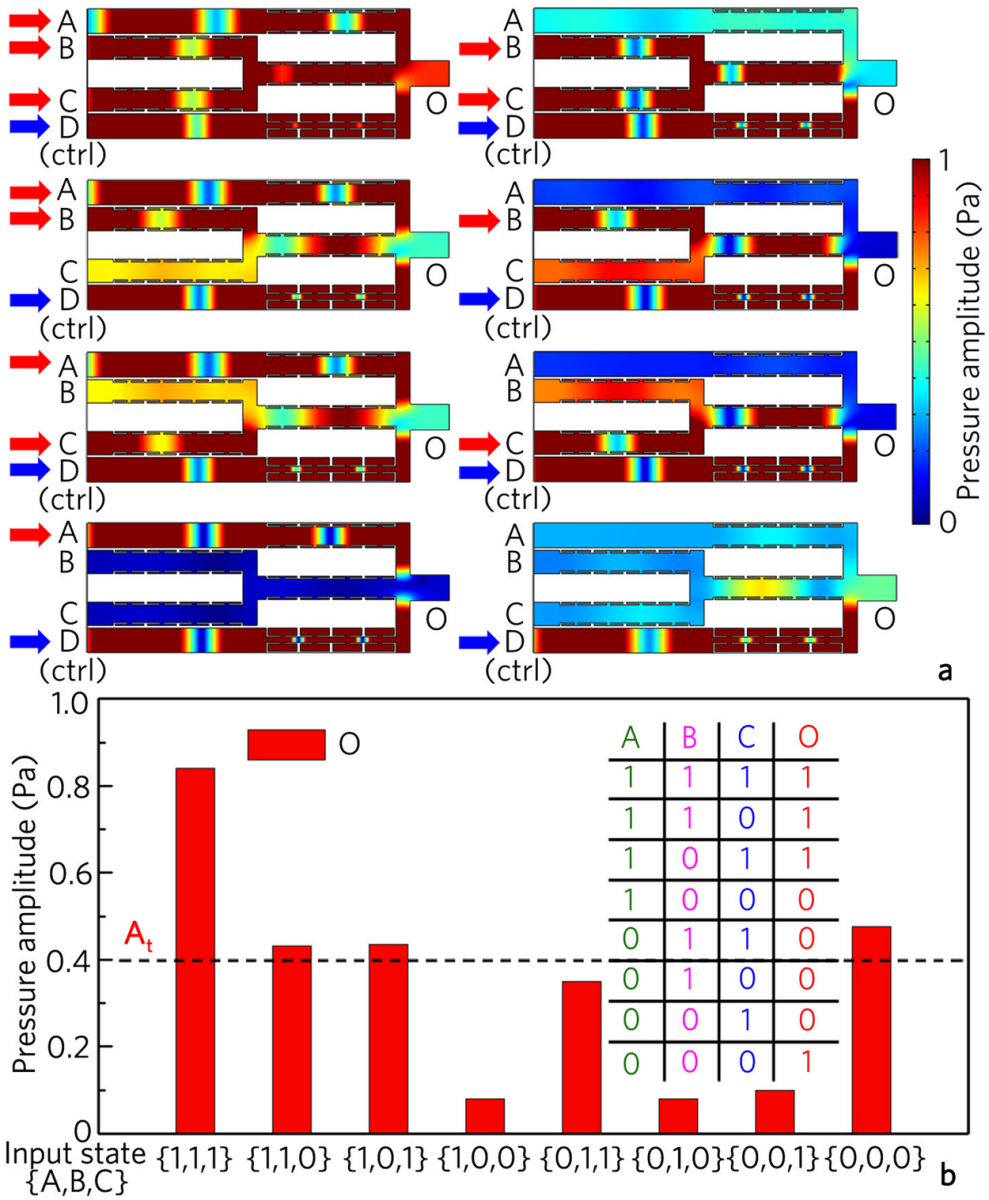
Acoustic logic operations. (**a**) Distributions of pressure amplitude field for logic operation A⊙(B+C) with different input states, and (**b**) corresponding output pressure amplitudes and true table.

We can also carry out complex acoustic logic operations based on the designed logic gates. As an example, we realize the logic operation A⊙(B+C) based on the logic gates OR and XNOR (shown in Fig. 13), in which A, B, and C are the input ports, and D is the control port. As shown in Fig. 13a, the output amplitudes at the port O are smaller than 0.4 Pa for the input states {1, 0, 0}, {0, 1, 1}, {0, 1, 0} and {0, 0, 1}, but are larger than 0.4 Pa for {1, 1, 1}, {1, 1, 0}, {1, 0, 1}, and {0, 0, 0}, which is also attributed to the linear interference phenomena of the three input signals and the control signal. Figure 13b shows the corresponding output amplitudes and the true table for the logic operation A⊙(B+C). With the uniform threshold of 0.4 Pa, the output states are {1}, {1}, {1}, {0}, {0}, {0}, {0} and {1} for the input states{1, 1, 1}, {1, 1, 0}, {1, 0, 1}, {1, 0, 0}, {0, 1, 1}, {0, 1, 0}, {0, 0, 1} and {0, 0, 0}, respectively. The output states agree well with the theoretical results of the logic operation A⊙(B+C). However, the output amplitudes for the input states {1, 1, 0}, {1, 0, 1} and {0, 1, 1} are close to the threshold. This is because the output amplitude of the logic gate OR is smaller than the standard input amplitude (1.0 Pa) of the XNOR gate for {1, 1, 0} and {1, 0, 1}, but is larger than 1.0 Pa for {0, 1, 1}. Practically, this challenge may be overcome by placing an acoustic triode structure at the junction of two logic gates, and the output saturation value of the acoustic triode is set as the standard input value of the lower level logic gate to normalize the output value of the upper level logic gate.

### Working bands of composite logic gates and logic operation

**Figure 14:**
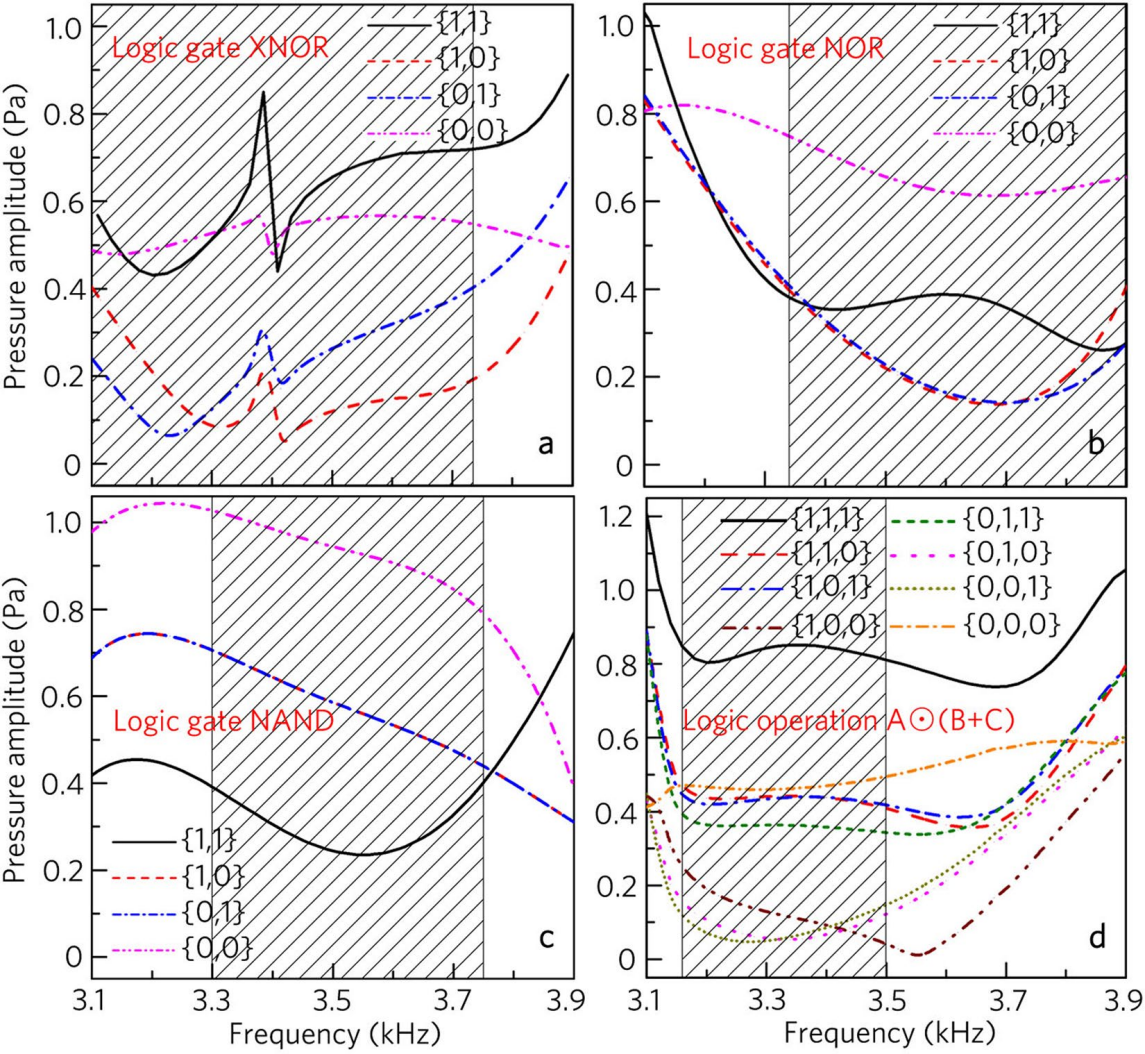
Working bands of composite logic gates and logic operation. Output amplitude spectra induced by composite logic gates (**a**) XNOR, (**b**) NOR, (**c**) NAND and (**d**) logic operation A⊙(B+C) for different input states.

Finally, we discuss the bandwidth of the composite logic gates XNOR, NOR and NAND and the logic operation A⊙(B+C), which is shown in Fig. 14. Compared with the working bands of the basic logic gates (shown in Fig. 6), the band of the logic gate XNOR (3100–3740 Hz) remains almost unchanged, but those of the logic gates NOR (3350–3900 Hz) and NAND (3300–3750 Hz) and the logic operation A⊙(B+C) (3160–3500 Hz) change greatly. This is because the logic functions NOR and NAND and the logic operation are realized based on two different logic gates, and there exists a mismatch between the output and input amplitudes of both the logic gates.

## Conclusion

In conclusion, we have demonstrated the binary-phase acoustic logic gate based on the multi-port waveguide structure, in which the width and length of the logic gate are only about 0.3λ and 0.6λ. The results show that the basic logic gates OR, NOT and AND, and the composite logic gate XOR with a uniform threshold of 0.4 Pa are realized based on the linear acoustic interferences, which arises from the phase manipulations of the binary-phase unit cells. The measured results agree well with the simulated ones, and the fractional bandwidth can reach about 0.2. Moreover, we design the composite logic gate XNOR by using the four-port waveguide, and realize the composite logic gates NOR and NAND and the logic operation A⊙(B+C) based on two different logic gates, corresponding to the logic gates NOT and OR, the logic gates NOT and AND, and the logic gates OR and XNOR, respectively. The proposed acoustic logic gates have advantages of the subwavelength size, broad bandwidth, and passive structure, which have important potential applications in acoustic computing, acoustic information processing and integrated acoustics.

## Methods

### Sample and experimental setup

The designed acoustic logic gates are fabricated with epoxy resin by three-dimensional printing technology. To obtain the same input signals, two same cylindrical acoustic sources are placed on the right side of the input ports, and the distance between the unit cell and the cylindrical source is 5 mm. The input signals are generated from the cylindrical sources driven by a power amplifier. On the left side of the output port, a 1/4 inch microphone (Brüel & Kjær type-4961) is adopted to measure output signals. The measured data is recorded by the Brüel&Kjær 3160-A-022 module, and is analyzed by the software PULSE Labshop.

### Numerical simulations

The finite element method (COMSOL Multiphysics software 5.2a) is utilized to numerically simulate the characteristics of the acoustic logic gates. The pressure field is calculated in the Acoustic-Solid interaction module, and the Helmholtz resonator arrays filled with air are fabricated with epoxy resin to satisfy the acoustic-structure boundary conditions. In addition, the boundaries of the waveguides are set as the sound hard boundary, and those of the input and output ports are the plane wave radiation boundary. The parameters used for air under an ambient pressure of 1 atm at 20 °C are mass density *ρ*_air_ = 1.21 kg/m^3^ and sound speed *c*_air_ = 343 m/s. The mass density, the longitudinal wave velocity and the transversal wave velocity for epoxy resin are 1180 kg/m^3^, 2720 m/s and 1460 m/s, respectively.
